# Breaking down prefixed words is unaffected by morphological boundary opacity: Evidence from behavioral and MEG experiments

**DOI:** 10.3758/s13423-025-02758-7

**Published:** 2025-08-12

**Authors:** Dave Kenneth Tayao Cayado, Samantha Wray, Marco Chia-Ho Lai, Adam J. Chong, Linnaea Stockall

**Affiliations:** 1 Department of Psychology, Royal Holloway, University of London, Egham, UK; 2 Department of Linguistics, Queen Mary, University of London, London, UK; 3 Department of Linguistics, Dartmouth College, Hanover, New Hampshire USA; 4 Department of Psychology, New York University, New York, NY USA

**Keywords:** Morphological decomposition, Visual word recognition, Magnetoencephalography (MEG), Masked priming, Morphophonology, Tagalog

## Abstract

Previous experiments support an initial stage of early, form-based visual word recognition, where morphologically complex words like *adorable* are segmented into morphemes {adore}+{-able}, despite an orthographic change in the stem. However, most experiments have focused on words with clear boundaries between the affix and stem, making decomposition more straightforward. We investigate whether obscured boundaries between the prefix and stem affect morphological decomposition. Using Tagalog as a test case, we compare the processing of prefixed words [1] without morphophonological changes (e.g., {ma**ng**}+{hila} becomes *ma****ng****hila* “to pull”), [2] with nasal assimilation obscuring prefix identity (e.g., {ma**ng**}+{bulag} becomes *ma****m****bulag* “to blind”), and [3] with nasal substitution obscuring both prefix and stem identities and their morphological boundary at orthographic and phonological levels (e.g., {ma**ng**}+{**t**ulak} becomes *ma****n****ulak* “to push”). Crucially, these morphophonological changes exhibit variability: nasal substitution is more likely than assimilation for voiceless-initial stems, while the opposite holds for voiced-initial stems. Experiment 1 presents behavioral masked priming data that prefixed words are decomposed into morphemes, even with obscured {prefix}+{stem} boundaries. Experiment 2 further supports these results with data from magnetoencephalography showing neural activity is modulated by stem:whole word transition probability, which indicates morphological decomposition. Findings from both experiments unambiguously show that early, form-based decomposition is robust and flexible enough to recognize morphemes, despite morphophonological changes obscuring the {prefix}+{stem} boundary.

**Supplementary Information:**

The online version contains supplementary material available at 10.3758/s13423-025-02758-7.

## Introduction

More than forty years of research provides converging evidence that morphologically complex words are segmented in the initial stage of visual word recognition, and analyzed in terms of their constituent morphemes (e.g., teacher ➔ {teach} + {-er}). Much of the evidence comes from masked priming experiments, where, for example, *teacher* significantly primes *TEACH* due to their morphological relationship (Rastle & Davis, 2008). However, the majority of these studies have focused on cases with a clear, transparent orthographic boundary between affix and stem, possibly making decomposition more straightforward (see Rastle and Davis (2008) for a review of suffixation studies and Stockall et al. (2019) for prefixation). Whether decomposition takes place in cases when the stem-affix boundary is orthographically and phonologically obscured remains a question. We address this question in this study by using Tagalog as a test case, where orthographic and phonological changes obscure stem and affix identities, and the boundary between them.

In addition to behavioral masked priming experiments, findings from magnetoencephalography (MEG) studies support early decomposition. MEG studies find that the M170 response from the Visual Form Word Area (VWFA)^1^ reliably tracks stem:whole word transition probability (TP) (the ratio between the frequency of the lexical stem to the frequency of the whole word), a variable argued to index decomposability and morphological complexity (Hay, 2001). Specifically, studies have found a significant correlation between TP and the M170 across different languages and morphological patterns (English: Gwilliams & Marantz, 2018; Lewis et al., 2011; Solomyak & Marantz, 2010; Stockall et al., 2019; Greek: Neophytou et al., 2018; Tagalog: Cayado et al., 2024; Wray et al., 2022). In most cases, the correlation between TP and the M170 is positive: as TP increases and as a whole word becomes more likely to be a continuation of (to transition from) a stem, the M170 response increases (but see General discussion for the issue of directionality). Overall, finding that the brain is sensitive to stem:whole word TP is taken to indicate that participants are sensitive to morphological structure within complex words, supporting models that assume early decomposition (Rastle et al., 2004; Stockall & Marantz, 2006).

Previous studies, however, have mainly examined written words that are perfectly segmentable (that is, *teacher* is segmented into *teach* and -*er*). Few studies have examined whether early decomposition remains robust for written words that are not perfectly segmentable due to orthographic and/or phonological changes. McCormick et al. (2008) found robust masked priming effects for English word pairs with orthographic obscurity due to a deleted “e” in the stem (e.g., *adorable-ADORE*). Tang and Witzel (2020) also found robust masked priming effects for phonologically obscured word pairs (e.g., *health-HEAL*, where [hɛl] and [hiːl] are allomorphs). These two studies suggest that neither orthographic nor phonological changes disrupt decomposition in visual word recognition in English. However, this is not the case in other languages. Frost et al. (2000) investigated the case of weak roots in Hebrew (e.g., *hpyl-HSPYK*, where *n* is deleted from the root *N-P-L* in the prime /hipil/ “he overthrew”)^2^ using a masked priming paradigm. This orthographic and phonological change in the consonantal root disrupted priming effects, thereby suggesting that the root may need to be fully recognizable to license decomposition in Hebrew.

The writing system differences between English and Hebrew may explain these mixed results. Hebrew roots typically only consist of consonants, so deleting a consonant in a triconsonantal root may be too disruptive for recoverability and recognizability, as the remaining consonants in the root can match with multiple other roots in the language. For example, the remaining *P-L* from *N-P-L* can match with other roots in Hebrew, such as *K-P-L* (e.g., *hkpyl */hikpil/ “to multiply”) and *P-L-G* (e.g., *pylg */pilug/ “division”). On the other hand, English roots remain recognizable and recoverable despite “e” deletion, since the remaining letters can still be easily mapped onto the full letter strings. For example, skilled readers of English can still recognize *adore*, despite the deleted final “e” in *ador*, since the remaining letter strings cannot be confused with any other roots in the language. Perhaps, deleting a consonant in Hebrew is much more disruptive than losing an unpronounced vowel, or changing the pronunciation of a vowel in English. These mixed results warrant further investigation of possible effects of orthographic and/or phonological changes on decomposition.

Although the previous studies in English involved cases where orthographic or phonological change prevented perfect segmentability, these words still have a relatively clear orthographic boundary between {stem}+{affix}. What remains unknown is whether decomposition is affected when the stem-affix boundary becomes obscured due to morphophonological changes – that is, phonological changes that are conditioned by the presence of a particular morpheme. Tagalog, an Austronesian language from the Philippines, offers an important test case. Affixing velar nasal-final prefixes like /maŋ-/ to obstruent-initial stems can trigger two changes: nasal **assim**ilation, which obscures the identity of the prefix in the phonology and the orthography without obscuring the word boundary (e.g.,/ma**ŋ**-/+/búlag/ becomes [ma**m**búlag] “to blind”), and nasal **subs**titution, which obscures both prefix and stem identities as well as the morphological boundary between them phonologically and orthographically (e.g.,/ma**ŋ**-/+/**t**úlak/ becomes [ma**n**úlak] “to push”). For the remainder of the paper, Assim and Subs refer to both phonological and orthographic levels of transparency in Tagalog, and all examples given are in orthographic forms using curly brackets for morpheme combinations and italicization for full affixed forms.

Crucially, whether a prefix-stem combination undergoes **assim** or **subs** is highly variable across the lexicon, but is partly phonologically predictable – **subs** is more likely than **assim** for voiceless-initial stems (*p, t, s, k*), while **assim** is more likely than **subs** for voiced-initial stems (*b, d, g*) (see Zuraw (2010) for formal analysis). This variability may add another level of processing difficulty when detecting the morphological boundary between prefix and stem, since the second nasal in *ma****m****-* could belong to the prefix or the stem (**assim:** *ma****m-****bulag* “to blind”; **subs:** *ma-****m****angka* “to ride a boat”).

Tagalog, therefore, allows us to investigate two related questions: [1] whether decomposition occurs even when the prefix identity is obscured due to nasal assimilation and when the prefix-stem boundary is obscured due to nasal substitution; and [2] how predictability of phonological patterns affects decomposition. Two experiments were conducted to address these questions. Experiment 1 used a behavioral lexical decision with a masked priming paradigm, while Experiment 2 used a single word lexical decision paradigm with concurrent MEG recording.

## Experiment 1

Experiment 1 investigated whether morphological decomposition occurs for morphologically complex words with obscured prefix identity due to nasal** assim**ilation and obscured morphological boundary due to nasal **subs**titution. If morphological identity matters, then priming effects elicited in both **subs** and **assim** should be reduced compared to those elicited by prefixed words that do not involve any changes (NoChange). However, if morphological decomposition is more difficult when the morphological boundary is obscured, then priming effects elicited by prefixed words in **subs** should be reduced compared to the prefixed words in **assim** and prefixed words in **NoChange**. If predictability of phonological patterns modulates decomposition, then **subs-Voiceless** should evoke larger priming than **Subs-Voiced**, as substitution is more likely for voiceless-initial stems than voiced-initial stems. Finally, if morphological decomposition is unaffected by obscured morphological boundary, then NoChange, Assim, and Subs should evoke significant priming of similar magnitude.

### Participants

Eighty native speakers of Tagalog were recruited for the experiment via Prolific.co (mean age =34.05 years, SD = 9.402, range = 20–57 years). Participants reported their city of origin in the Philippines. Only participants from cities where Tagalog is the dominant language were retained. Due to the multilingual nature of the Philippines, all participants also reported speaking English. Others additionally spoke another Philippine language (*N =* 53), Mandarin Chinese (*N =* 5), Spanish (*N =* 4), German (*N =* 3), Italian (*N =* 1), Vietnamese (*N =* 1), and Greek (*N =* 1). All participants reported doing their primary and secondary education in the Philippines. The experiment was overseen by the first author’s university human subjects’ ethics review board. All participants gave their informed consent and were remunerated £3.13 for the 30-min experiment.

### Design and materials

**Fig. 1:**
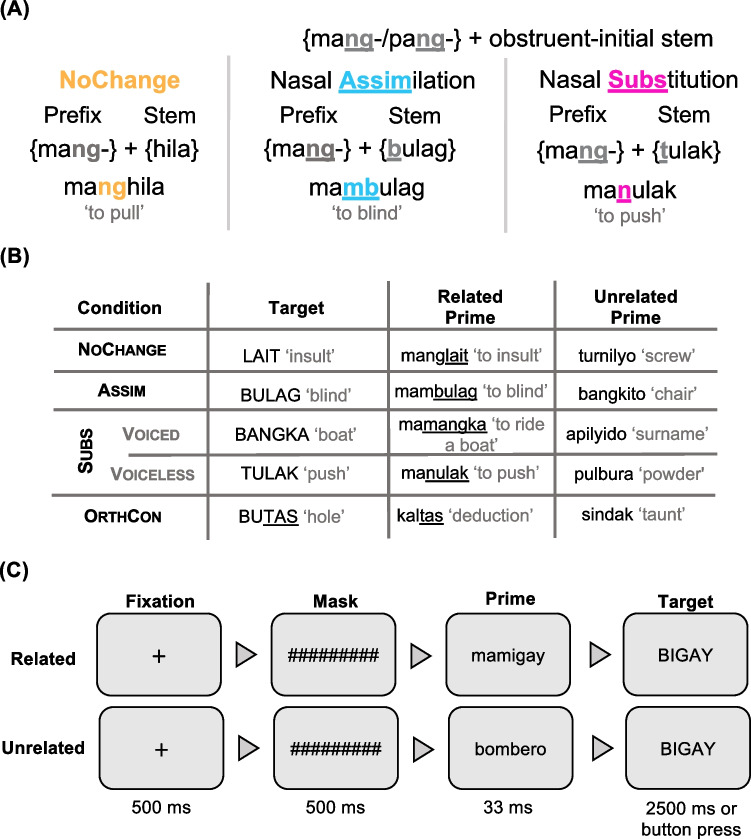
Experimental design. **(A)** examples of nasal assimilation and nasal substitution. **(B)** Sample items for each condition. **(C)** Sample trial in the experiment

The experiment exploits the processes of nasal **assim**ilation and nasal **subs**titution in Tagalog. As described above, three prefixes with a final velar nasal (*mang-, pang-,* and *nang-*) can undergo and trigger phonological processes when attached to obstruent-initial stems. In some cases, **assim** occurs, where the prefix-final velar nasal takes on the place of articulation of the initial sound of the stem. For example, the prefixes *pa****ng***-*, ma****ng***-*,* and *na****ng***- surface as *pa****m****-/ma****m****-/na****m****-* when combined with *p-*initial and *b*-initial stems, and as *pa****n****-/ma****n****-/na****n****-* when combined with *d-, t-,* and *s-*initial stems. **assim** is also triggered by liquid sonorants, surfacing as *pa****n****-/ma****n****-/na****n****-* for *l-* and *ɾ-*initial stems. The form of the stem remains unchanged during nasal assimilation, therefore, the morphological boundary between prefix and stem is unaffected in both orthography and phonology (see Fig. 1A).

The same three prefixes can instead trigger **subs**, where the prefix-final velar nasal and the initial obstruent of the stem are both replaced by a nasal sound with the same place of articulation as the stem’s original obstruent (Schacter & Otanes, 1972; Zuraw, 2010). For example, the velar nasal in *mang-* and the initial consonant in *tulak* “push” become one sound *n* in *manulak* “to push someone.” Both the prefix and the stem form change, and the morphological boundary between prefix and stem is obscured in both the orthography and the phonology. In other words, the phonological changes are reflected transparently in the orthography of the language.

Crucially, whether a prefix-stem combination undergoes **assim** or **subs** is highly variable across the lexicon, but is partly phonologically predictable (Zuraw, 2010). Specifically, **subs** is more likely than **assim** for voiceless-initial stems (e.g., *p, t, s, k*), while **assim** is more likely than **subs** for voiced-initial stems (e.g., *b, d, g*), but exceptions to these generalizations exist (e.g., {ma**ng**}+{**b**angka} = *ma****m****angka* “to drive a boat” via **subs**).

We used a visual masked morphological priming paradigm with three critical conditions with 40 trials each (20 related, 20 unrelated):

1. **NoChange**, consisting of prefixed words with no morphophonological changes (*mang*-, *pang*- prefixes attached to *h-*initial, *y-*initial, and *nasal-*initial stems);
2. **Assim**, consisting of nasal-assimilated prefixed words; and
3. **Subs**, consisting of nasal-substituted prefixed words.

Further, the Subs condition was divided into two sub-conditions, with 20 trials each (10 related, 10 unrelated): **Subs-Voiced** condition, which consisted of nasal-substituted prefixed words with voiced-initial stems, and **Subs-Voiceless** condition, which consisted of nasal-substituted prefixed words with voiceless-initial stems. The 20 target words in the critical conditions were preceded either by a morphologically related prefixed prime or a morphologically unrelated prime. The prefixes *mang-* and *pang-* were included in all critical conditions. We excluded the prefix *nang-*, as it is the perfective form of the prefix *mang-*.

An orthographic control (**OrthCon**) condition also had 40 trials (20 related, 20 unrelated). Orthographic relatedness was operationalized as second syllable overlap, to replicate the orthographic overlap in critical conditions. Figure 1B shows example items for each condition. There were 160 critical trials (80 related, 80 unrelated) in this experiment. Ten practice trials were also added, consisting of five real prefixed words and five nonwords that were not used in the test phase.

Stimuli were selected from a 198,303,250-word Tagalog corpus from SketchEngine (Kilgarriff et al., 2004), an online platform that compiles web-based text corpora from more than 900 languages. We generated a candidate set of 1,132 items with *mang-* (*mam-, man-, mang-*) and *pang-* (*pam-, pan-, and pang-*) prefixes. We then restricted the candidates to obstruent-initial stems that Zuraw (2010) showed to exhibit Assim and Subs, and retained items with sonorant-initial stems to have items where *pang*- and *mang-* do not show morphophonological variation. We excluded items that exhibit two morphological processes (prefixation and reduplication, e.g., {pang}+{RED}+{kahoy} “wood” = *pangangahoy*), as these words might trigger more complex morphological processing mechanisms. Finally, we excluded candidates with alternative pronunciations and spellings in the corpus (e.g., {pang}+{tuhog} “stick” = *pa****n****uhog* (Subs) or *pa****nt****uhog* (Assim), as we are interested in the variability of phonological patterns across the lexicon, not variability at the individual word level.^3^ These exclusion criteria left us with 252 candidates. To ensure these candidates have a stable pronunciation and orthography, a forced-choice decision-norming task was created in which native Tagalog speakers had to choose which is acceptable between two pairs: e.g., *mampaso* “to burn” (Assim) and *mamaso* “to burn” (Subs) for {mang}+{paso}. This norming task was implemented in Gorilla.sc, and 30 Tagalog-speaking participants were recruited via Prolific.co. Only those words that were consistently accepted as either nasal-assimilated or nasal-substituted were retained, resulting in 105 items. For the OrthCon condition, following the procedure outlined in Cayado et al. (2023), we first created a set of word pairs with the same second syllable. We also created a set of unrelated word pairs, which have no semantic and very minimal orthographic overlap between each other as established by Levenshtein score (Levenshtein, 1965). The targets in the NoChange, Assim, and Subs conditions were list-wise matched for orthographic length and stem frequency (see Table 1). Related and Unrelated primes were pairwise matched for orthographic length and list-wise matched for lexical frequency. The same matching was employed in the OrthCon condition.

**Table 1:** Mean item characteristics for all sets and prime

Condition	Per million frequency	Letters	Levenshtein score
**NoChange**			
Target	21.18	4.75	
Related Prime	1.50	8.75	
Unrelated Prime	1.42	8.75	
**Assim**			
Target	23.05	4.95	
Related Prime	1.57	7.95	
Unrelated Prime	1.63	7.95	
**Subs**			
Target	21.77	5.60	
Related Prime	1.85	7.65	
Unrelated Prime	1.85	7.65	
**OrthCon**			
Target	21.86	5.00	
Related Prime	8.31	6.10	2.90
Unrelated Prime	8.23	6.10	5.45

Nonwords were generated with the Wuggy toolkit augmented with a Tagalog wordlist as training data (Keuleers & Brysbaert, 2010). A total of 160 nonword targets and 80 nonword primes were included as filler trials. All nonwords were pronounceable and five to ten letters long to match the critical and control trials. Half of the nonword targets (80) were preceded by six- to ten-letter long real word primes to avoid cueing participants to the lexical decision. Both nonword and real word primes were list-wise matched for orthographic length. There was a total of 330 trials in this experiment.

### Procedure

The experiment was administered on participants’ computers using Gorilla (www.Gorilla.sc) (see Cayado et al. (2023) and Angele et al. (2022) for the validity of online experiment presentation platforms for masked priming experiments). We presented the stimuli in two equal blocks of 160 trials (320 critical trials in total), preceded by ten practice trials. All participants saw the critical and control targets in both related and unrelated prime contexts. Targets were only seen once per block, and the blocks were interrupted by a short break to minimize long-lag priming effects. The order of items within a block was pseudo-randomized for each participant, and the order of the two blocks was counterbalanced across participants such that each block occurred as the first block for half of the participants.

All visual stimuli were presented in the center of the screen, in black 50-point size Courier Sans Serif against a white background. Each trial began with a fixation cross for 500 ms, followed by a 500-ms presentation of a forward mask of hash marks (e.g., ##########), matching the maximum prime length. Immediately afterward, the lowercase prime was displayed for 33 ms, followed by the UPPERCASE target. Participants performed a lexical decision task by judging whether the target word was a real Tagalog word or not by pressing the corresponding arrow key (see Fig. 1C). The target word disappeared after 2,500 ms if no response was recorded. For half of the participants, “Oo” (Yes) mapped to the left arrow key and “Hindi” (No) mapped to the right arrow key, while the other half experienced the opposite mapping. Button-press counterbalancing was independent of block order. Participants were instructed to respond as quickly and as accurately as possible. We further probed participants’ perceptions of the primes after the task. None of the participants reported noticing the masked primes.

### Data analysis

All analyses were performed using R 3.6.1 (R Core Team, 2019). Since this experiment was conducted online, stimulus presentation may be subject to delay. Hence, we first checked whether the primes were, in fact, masked. Participants who saw any primes for > 60 ms were removed, leading to the exclusion of 13 subjects or 16.25% of the data. We then checked for error rates on the responses to target words, and participants with an accuracy rate of < 60% were excluded, which led to the removal of four participants or 5% of the data. Sixty-three participants were retained for further analysis. For the reaction time (RT) analysis, RTs faster than 200 ms were removed (0.24%) (i.e., minimal trimming). We followed Baayen and Milin (2010) for further outlier trimming; we fit a simple mixed model with only random effects and excluded all data points with residuals exceeding 2.5 SD (2.53%). Incorrect responses were then excluded (6.73%). A total of 28.75% of the data was excluded during the cleaning process. 9,180 data points were retained for further analyses (NoChange: 2329; Assimilation: 2269; Substitution: 2300 (Subs-Voiced: 1119; Subs-Voiceless: 1181); OrthCon: 2222). See Table 2 for by-subject mean RT and mean accuracy in each condition.

**Table 2:** By-subject mean reaction times (RTs) in ms, priming effects, and accuracy scores for all conditions and prime types

Prime type	Mean RT (ms)	Priming effect (ms)	Accuracy (%)
**NoChange**			
Morph.Rel	757.69	17.85	94.04
Morph.Unrel	775.54		93.65
**Assim**			
Morph.Rel	778.87	17.34	91.42
Morph.Unrel	796.21		91.42
**Subs**			
Morph.Rel	766.53	23.64	92.22
Morph.Unrel	790.17		93.17
**OrthCon**			
Orth.Rel	792.69	−9.044	89.92
Orth.Unrel	783.65		89.44

Accuracy rates and cleaned RTs were analyzed using (generalized) linear mixed-effects modeling as implemented in the *lme4* package (v1.1-21; Bates, Maechler, et al., 2015). The models included Condition (NoChange, Assim, Subs, OrthCon) and PrimeType (Related, Unrelated) as fixed effects and their interaction. We also included the lexical properties target and prime length in letters, and prime and target frequency as fixed effects. We set NoChange and Related as reference levels in the Condition and PrimeType conditions, respectively. To test whether the variability of phonological patterns affects decomposition, we ran further mixed-effects model analyses only containing items from the Subs condition divided into two sub-conditions: Subs-Voiceless, with voiceless-initial stems, and Subs-Voiced, with voiced-initial stems. The fixed effects were StemC (Subs-Voiceless, Subs-Voiced) and PrimeType (Unrelated, Related), as well as their interactions. Log-likelihood ratio tests using the *anova()* function were used for significance testing, comparing models with and without the interaction (Condition * PrimeType vs. Condition + PrimeType). Multiple comparison correction was done using the *emmeans* package in R (Lenth et al., 2022) and alpha was.05.

We tested for the inclusion of by-participant and by-target random slopes for the Condition*PrimeType interaction (Matuschek et al., 2017), but convergence issues emerged, prompting us to simplify the model. The final model included only random intercepts:

$$\text{RT }\sim \text{CONDITION} \times \text{PRIMETYPE}+\text{PRIMELENGTH}+\text{PRIMEFREQ}+\text{TARGETLENGTH}+\text{TARGETFREQ}+\left(1\left|\text{Participation}\right.\right)+\left(1\left|\text{Target}\right.\right)$$

we focus below on the RT data; accuracy results are presented in full in the Online Supplementary Materials  on the Open Science Framework (OSF) link.

### Results and discussion

**Fig. 2:**
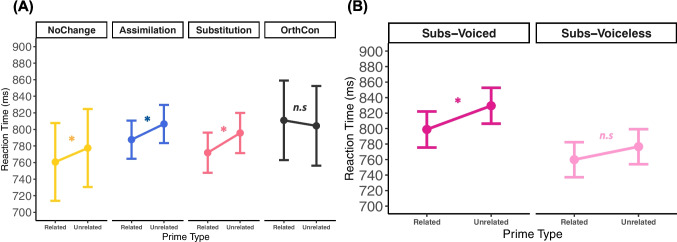
The magnitude of priming effects was computed using the formula RT^Related Prime^ – RT^Unrelated Prime^. **(A)** Priming effects in milliseconds (ms) by condition, illustrating that all morphologically related prime-target pairs evoked priming, in contrast with orthographic overlap prime-target pairs. **(B)** Priming effects in ms for Subs-Voiced and Subs-Voiceless conditions

For the RT analysis, mixed-effects modeling revealed a significant interaction between PrimeType and Condition (*X*^2^(4) *=* 11.83, *p* = 0.0079), suggesting that the RT difference between related and unrelated primes differed depending on Condition. There was also a significant main effect of PrimeType [*X*^2^(4) *=* 15.74, *p* = <.00001], and TargetFreq [*X*^2^(4) *=* 8.83, *p* = 0.0029]. No other fixed effect reached significance (see Table 3). Planned pairwise comparison of related and unrelated RT means per condition using Tukey’s HSD tests showed that significant priming effects were obtained only for the critical conditions (NoChange, *estimate* = 16.83, SE = 6.77, z = 2.520, *p* = 0.0117; Assim, *estimate* = 18.94, SE = 6.77, z = 2.798, *p* = 0.0051; Subs, *estimate* = 23.79, SE = 6.72, z = 3.540, *p* = 0.0004), not the OrthCon condition (*estimate* = −6.59, SE = 6.84, z = −0.963, *p* = 0.3355). The differences between NoChange-OrthCon (*estimate* = 23.42, *SE* = 9.56, 95% CI = [4.68, 42.16], *t* = 2.45, *p* = 0.0143), Assim-OrthCon (*estimate* = 25.53, *SE* = 9.62, 95% CI = [6.67, 44.40], *t* = 2.65, *p* = 0.0080), and Subs-OrthCon (*estimate* = 30.38, *SE* = 9.59, 95% CI = [11.58, 49.18], *t* = 3.16, *p* = 0.0015) were all significant. No other pairwise differences were significant (see Fig. 2).

**Table 3:** Reaction time mixed-effects model summary

Formula: RT ~ Condition * PrimeType + PrimeLength + PrimeFreq + TargetLength + TargetFreq + (1|Participant) + (1|Target)				
**Fixed effects:**	**Estimate**	***df***	***t* value**	**Pr(>|*t*|)**
(intercept)	720.22	79.62	5.74	<.0001***
Condition = Assim	29.04	84.22	0.88	0.381
Condition = Subs	18.16	78.12	0.30	0.761
Condition = OrthCon	26.82	77.49	0.30	0.762
PrimeType = Related	−16.83	8981.08	−2.52	0.011*
PrimeLength	5.31	77.34	0.18	0.857
PrimeFreq	0.37	90.10	0.33	0.737
TargetLength	7.22	77.27	0.24	0.808
TargetFreq	−0.94	75.67	−3.06	0.003**
Interaction, PrimeType:Condition = Assim	−2.10	8982.82	−0.22	0.824
Interaction, PrimeType:Condition = Subs	−6.95	8979.88	−0.73	0.462
Interaction, PrimeType:Condition = OrthCon	23.42	8980.17	2.45	0.014*
Significant codes: 0 “***” 0.001 “**” 0.01 “*” 0.05 “.” 0.1 “ “ 1				
**Random effects:**	**Variance**			
Participant	14682			
Target	2105			
Residual	25961

We then tested the effects of voicing in the Subs condition (Fig. 2B). We did not find a significant interaction between StemC and PrimeType (*X*^2^(4) *=* 1.13, *p* = 0.2872), but there were significant main effects of PrimeType (*X*^2^(4) *=* 12.86, *p* = 0.0003) and StemC (*X*^2^(4) *=* 6.59, *p* = 0.0102). Planned pairwise comparison further revealed that only Subs-Voiced items exhibited significant priming (*estimate* = 30.7, SE = 9.38, z = 3.269, *p* = 0.0011), while Subs-Voiceless showed a non-significant priming effect (*estimate* = 16.7, SE = 9.13, z = 1.834, *p* = 0.0667). The priming effect difference between Subs-Voiced and Subs-Voiceless conditions, however, was not significant (*estimate* = 13.93, SE = 13.09, 95% CI = [−39.59, 11.72], *t* = −1.06, *p* = 0.2872) (see Online Supplementary Materials for Bayesian analysis corroborating this frequentist analysis).

Overall, this experiment provides evidence that prefixed words in NoChange, Assim, and Subs conditions are decomposed into morphemes, suggesting that boundary and identity opacity due to variable morphophonological changes do not hamper early decomposition. Further analysis of the Subs-Voiceless and Subs-Voiced sub-conditions suggests that the variability in nasal substitution application does not seem to modulate decomposition. However, the failure to find any effect of morphological boundary obscurity may be due to behavioral masked priming not being sufficiently sensitive, since it reflects not just the initial decomposition that happens ~200 ms after seeing the word, but also subsequent lexical decision processes reflected in the button press ~800 ms later. It is possible that we failed to find any effect of morphological boundary and identity obscurity since this might only emerge at very early stages of morphological processing, i.e., before 200 ms. To isolate the initial decomposition phase, Experiment 2 used MEG which allows us to focus our analysis on the first 200 ms of seeing a written word.

## Experiment 2

Using a single word lexical decision paradigm with concurrent MEG recording, we further investigated how morphological boundary opacity due to morphophonological changes affects the way prefixed words are decomposed. Using MEG allows us to more narrowly focus our investigation on the first 200 ms of seeing a written word (Zweig & Pylkkänen, 2009), which is when early decomposition occurs. If prefixed words in NoChange, Assim, and Subs are fully decomposed, then we expect to find a significant correlation between stem:whole word transition probability (TP) across the three crucial conditions and the M170 activity in the VWFA, as identified by the functional localizer used in Cayado et al. (2024). These findings would be consistent with previous MEG experiments showing a significant correlation between TP and M170 activity (Gwilliams & Marantz, 2018; Lewis et al., 2011; Solomyak & Marantz, 2010; Stockall et al., 2019; Cayado et al., 2024; Wray et al., 2022). However, if identity changes disrupt early form-based decomposition, then only prefixed words in NoChange should exhibit a significant correlation between TP and the M170 activity. Further, if boundary obscurity affects decomposition, then prefixed words in the Subs condition should not evoke a robust TP/M170 correlation, but items in the Assim condition should pattern with NoChange.

### Participants

Twenty-three native Tagalog speakers (mean age = 36.7 years, SD = 10.021, range = 20–52 years) from the Filipino community in Abu Dhabi, United Arab Emirates, participated in this study. All participants were right-handed, with normal or corrected-to-normal vision. Five participants were removed from analyses due to noisy data (~20% of trials rejected during epoching and ICA, *n* = 1), low lexical decision accuracy task (*n* = 2), falling asleep during the experiment (*n* = 1), and reading the stimuli aloud (*n =* 1). Eighteen subjects were retained for analyses. The experiment was overseen by the ethics review board of the university where the data were collected.

### Design and materials

This experiment had the same critical conditions as Experiment 1: no-change (NoChange), nasal assimilation (Assim), and nasal substitution (Subs). Items in these conditions were taken from the same candidate list as Experiment 1. NoChange had 17 items, Assim had 18 items, and Subs had 20 items for a total of 55 critical items, which are list-wise matched as close as possible for length (in letters) and surface frequency (see Table 4 for summary). Critically, these items were coded for stem:whole word Transition Probability (TP). TP is the ratio between the frequency of the lexical stem/base to the frequency of the whole word, a variable argued by Hay (2001) to index decomposability (Gwilliams et al., 2016; Gwilliams & Marantz, 2018; Lewis et al., 2011; Solomyak & Marantz, 2010). The term “transition probability” reflects the fact that the closer the stem and the whole word are to having the same frequencies (a ratio closer to 1), the more probable the whole word is as a continuation of (transition from) the stem. Items in this experiment vary in base frequency and surface frequency, which ensures sufficient variation in TP across items. Further, lexical properties of these items were centered to reduce any multicollinearity issues. These items and their lexical properties were extracted from a 198,303,250-word Tagalog corpus from SketchEngine (Kilgarriff et al., 2004). Note that these materials were interleaved with those of another MEG experiment investigating morphological decomposition and recombination in Tagalog inflected words (Cayado et al., 2024), with 55 real prefixed words and 110 nonwords, which served as fillers for the current experiment. There were an equal number of real prefixed words and nonwords in the experimental session.

**Table 4:** Properties of items included as visual lexical decision stimuli in MEG experiment

Condition	Sample item	Average frequency (range) per million	Average TP (range)	Average length in letters
NoChange	ma**ng**hula “to guess”	0.507 (0.09, 1.50)	0.018 (0.0004, 0.0776)	5.11
Assim	ma**m**bulag “to blind”	0.578 (0.04, 1.94)	0.020 (0.0006, 0.0800)	4.88
Subs	ma**m**alo “to slap”	0.932 (0.07, 3.97)	0.023 (0.0009, 0.0830)	5.65

### Procedure

Stimuli were projected onto a screen that was located approximately 85 cm away from the subject using Presentation software (Version 18.0, Neurobehavioral Systems, Inc., Berkeley, CA, USA; www.neurobs.com).

Each trial started with a fixation cross at the center of the screen for 500 ms, followed by the stimulus for 2,500 ms or a button press (whichever came first), and ended with a blank inter-stimulus interval screen for 300–500 ms. Participants judged whether the word on the screen was a real Tagalog word or not by button press. For half the participants, the “Oo” (Yes) response mapped to the left button, and the “Hindi” (No) response mapped to the right button, with reverse mapping for the other half. Participants were instructed to avoid blinking to minimize MEG data artifacts. Items were fully randomized.

Data were continuously recorded at a sampling rate of 1,000 Hz using a 208-channel axial gradiometer whole-head MEG system (Kanazawa Institute of Technology, Kanazawa, Japan) and was filtered during acquisition between 0.1 Hz and 200 Hz. Participant headshape was digitized prior to recording using a hand-held FastSCAN laser scanner (Polhemus, VT, USA). Coils attached to predefined anatomical regions determined participants’ head position during the experiment. The head scan and the coil measures were then used for the co-registration process.

### Data preprocessing and analysis

Noise was removed from the raw data using eight gradiometer reference channels located away from the participant’s head and the Continuously Adjusted Least Squares Method (CALM; Adachi et al., 2001) in the MEG160 software (Yokohawa Electric Corporation and Eagle Technology Corporation, Tokyo, Japan). Subsequent preprocessing and analyses of the noise-reduced data were done using MNE-Python (Gramfort et al., 2013, 2014). The noise-reduced data were filtered using a low-pass infinite impulse response (IIR) 4th order Butterworth forward-backward filter with an upper cutoff frequency of 40 Hz. We then performed an Independent Component Analysis (ICA) on the filtered data to remove components corresponding to biomagnetic artifacts, like eye movements (blinks, saccades) and pulse. The data were epoched from −100 ms to 600 ms, relative to the stimulus onset. We then performed automated rejection of epochs with activity exceeding ± 2,000 fT/cm as well as visual inspection to reject epochs with noisy activity. On average, epoch rejection rate across trials is 0.86% (SD = 3.61%).

MEG data were co-registered using the FreeSurfer average brain (CorTechs Labs Inc., La Jolla, CA, USA) by manually scaling the participants’ digitized headshapes and the FreeSurfer average skull. A surface-based source space was constructed using an “ico-4” resolution, resulting in 2,562 vertices in each hemisphere. A boundary element model (BEM; Mosher et al., 1999) was used to compute the forward solution. A channel-noise covariance matrix was estimated using the 100-ms baseline intervals and regularized by the automatic method (Engemann & Gramfort, 2015). With the forward solution and noise covariance matrix, the inverse solution was computed using a fixed orientation (Gwilliams et al., 2016; Gwilliams & Marantz, 2018) and a signal-to-noise ratio (SNR) value of 2. The inverse solution was then applied to each artifact-free epoch and resulted in noise-normalized dynamic statistical parameter maps (dSPM; Dale et al., 2000).

We used an area within the left anterior fusiform gyrus, identified by a functional localizer from Cayado et al. (2024), as a functional region of interest (fROI) in this experiment. Cayado et al. successfully identify the Type II “string” response (also known as M170), a brain response associated with the perception of letter strings versus symbol strings peaking around 130–180 ms in the anterior portion of the left fusiform gyrus (Gwilliams et al., 2016; Tarkiainen et al., 1999). This activity has been demonstrated to correlate with stem:whole word TP, suggesting that this time window and region are sensitive to morphological complexity (Solomyak & Marantz, 2010). For each trial, the brain activity was extracted and averaged across the fROI and the time window between 130 and 180 ms. Single-trial data were then used as input for linear mixed effects regression (LMER) models using R (R Core Team, 2019) and *lme4* (Bates, Maechler et al., 2015). Specifically, we used LMER to investigate the effect of stem:whole word TP, as well as other lexical properties, on the dSPM averaged across the fROI and 130- to 180-ms time window. Initial fixed effects were Condition (NoChange, Assim, Subs), stem:whole word transition probability (TP), log base frequency (LogBaseFreq), log whole word frequency (LogWWFreq), and Length (in letters). We compared models with and without the fixed effect of interest through log-likelihood ratio tests using the *anova()* function, at an alpha level of.05. Only fixed effects that significantly improved the model BIC and AIC were retained in the final model. Selection of random effects proceeded via backward selection from the maximal model for both participant and item effects using the *lmerTest* package (for discussion, see Bates, Kliegl et al., 2018). We encountered convergence issues, prompting us to further simplify our model. The final model included a single random-intercept for participant:

$$\text{dSPM }\sim \text{CONDITION} \times \text{TP}+\text{LOGBASEFREQ}+\left(1\left|\text{Participant}\right.\right)$$

For the behavioral analysis, we first excluded the same five participants excluded from the MEG data. RTs faster than 200 ms were removed (0.16%) (i.e., minimal trimming). We followed Baayen and Milin (2010) for further outlier trimming; we fit a simple mixed model with a random effect for (1|Participant) and excluded all data points with residuals exceeding 2.5 SD (1.23%). Incorrect responses were excluded (8.01%). Three items were removed from both behavioral and MEG analyses due to having high TP values compared to the other items, resulting in a total of 52 critical items (17 for NoChange; 17 for Assim; 18 for Subs). We analyzed behavioral data using linear mixed-effects models. We focus below on the MEG data – behavioral results are presented in full in the Online Supplementary Materials on the OSF.

### Results and discussion

**Fig. 3:**
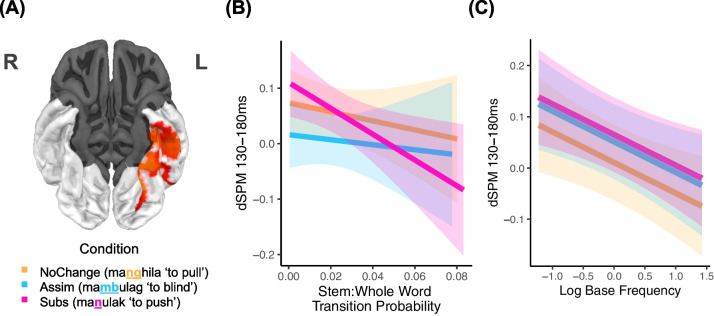
**(A)** functional region of interest. **(B)** Average activity from 130–180 ms in the left fusiform gyrus plotted against stem:whole word transition probability. **(C)** Average activity from 130–180 ms in the left fusiform gyrus plotted against log base frequency. Shaded areas represent the 95% confidence interval

Using the anterior fusiform fROI shown in Fig. 3A, we found no significant interaction between Condition and TP, *X*^2^(4) = 3.667, *p* = 0.159, but there was a significant main effect of TP, *X*^2^(4) = 9.528, *p* = 0.002, suggesting the effect of TP on dSPM did not differ across conditions. The effect of TP is plotted in Fig. 3B, showing that the relationship between TP and dSPM is negative for all conditions: as TP increases and it becomes more likely for a whole word to contain its stem, the evoked M170 decreases. Mixed-effects modelling also found a significant effect of LogBaseFreq (*X*^2^(4) = 5.195, *p* = 0.022). As shown in Fig. 3C, the relationship between LogBaseFreq and dSPM is also negative: the higher the base frequency, the smaller the evoked M170. No other lexical variables reached significance. See Table 5 for the model summary. In the RT data, we also found a significant effect of TP (*X*^2^(4) *=* 10.119, *p* = 0.001), supporting the assumptions of full decomposition models and replicating the findings of the online behavioral data in Experiment 1.

**Table 5:** Summary of the main LMER model showing correlation coefficients of lexical statistics and morphophonological types to source component amplitudes (left hemisphere)

Formula: dSPM ~ Condition * TP + LogBaseFreq + (1|Participant)				
**Fixed effects:**	**Estimate**	***df***	***t* value**	**Pr(>|*t*|)**
(intercept)	0.0106	29.77	0.284	0.778
Condition = Assim	0.0407	808.75	1.374	0.169
Condition = Subs	0.0543	808.93	1.860	0.063
TP	−1.3147	808.39	−1.295	0.195
LogBaseFreq	−0.0599	808.42	−2.629	0.008*
Interaction, TP:Condition = Assim	0.0237	808.50	0.019	0.985
Interaction, TP:Condition = Subs	−2.1794	808.66	−1.619	0.105
Significant codes: 0 “***” 0.001 “**” 0.01 “*” 0.05 “.” 0.1 “ “ 1				
**Random effects:**	**Variance**			
Participant	0.017			
Residual	0.115			

These MEG findings suggest that the brain is sensitive to morphological constituents within written words, regardless of morphological boundary and identity obscurity in both the orthography and the phonology. The negative correlation between TP and the M170 is consistent with that found for Tagalog infixation (Wray et al., 2022), but inconsistent with the direction found for English suffixation (Gwilliams & Marantz, 2018; Lewis et al., 2011; Solomyak & Marantz, 2010), Greek suffixation (Neophytou et al., 2018), and Tagalog reduplication (Wray et al., 2022) and prefixation (Cayado et al., 2024). We discuss this varying directionality below. Regardless of the directionality, the fact that there is a significant TP/M170 correlation is consistent with visual word recognition models that assume early decomposition (Rastle et al., 2004; Stockall & Gwilliams, forthcoming).

## General discussion

The main goal of the present study was to examine whether morphological decomposition still occurs for words that cannot be perfectly segmented into morphemes due to obscured prefix-stem boundaries. Experiment 1 found masked priming for prefixed words with no morphophonological changes, prefixed words with nasal assimilation, and prefixed words with nasal substitution. No reliable priming effects were obtained, however, for word pairs sharing non-morphological orthographic overlap, suggesting that the priming effects found in the critical conditions above are truly morphological. Although our results are consistent with previous findings that decomposition remains robust despite orthographic or phonological changes (McCormick et al., 2008; Tang & Witzel, 2020), the present study is different in three aspects: first, we examined an example that involves both orthographic and phonological changes/obscurity. Contrary to findings in Hebrew, where orthographic and phonological deletion of an initial consonant prevented decomposition, we show that decomposition is unaffected even when the changes affect both orthographic and phonological representations; second, the nasal substitution case we examined is more orthographically and phonologically obscured than previously investigated English cases (deleted “e”: *adorable-ADORE*; vowel change: *health-HEAL*), thereby attesting to the robustness of morphological decomposition; and finally, we provide evidence that decomposition remains intact and operational in the face of variable morphophonological changes, not just for predictable and categorical orthographic changes like in English (e.g., the final “e” in a stem is deleted when attached to a vowel-initial suffix).

Experiment 2 further examined early decomposition using a single-word lexical decision task with concurrent MEG. We found a significant negative correlation between stem:whole word TP and neural activity in the VWFA, but no interaction between TP and Condition, suggesting that all prefixed words are successfully segmented into morphemes, despite the obscured prefix-stem boundary. This negative correlation has also been previously found in Tagalog infixation (Wray et al., 2022): as it becomes more likely for a whole word to contain its stem, less neural activity is evoked. Previous MEG studies involving suffixation in English (Lewis et al, 2011; Solomyak & Marantz, 2010) and Greek (Neophytou et al., 2018), as well as prefixation (Cayado et al., 2024) and prefixal reduplication in Tagalog (Wray et al., 2022) found a positive correlation between TP and VWFA-based activity. Why then do we find a negative correlation in some cases, but a positive correlation in others? A full answer will require further research, but we note that the negative correlation is largely evoked by complex words with form disruption (infixation, nasal assimilation, and nasal substitution in Tagalog), with the strongest negative correlation being found in the Subs condition, wherein the morpheme boundary is most obscured. In contrast, words with no form disruption typically evoke a positive correlation. This positive correlation has been interpreted as indexing certainty – the closer the TP is to 1, the more certain the parse. But this TP measure is calculated based on the frequency of the actual stem in the lexicon. In contexts where the stem is not transparently available in the visual input, the more certain parse may be one where the stem is more probable given the whole word, i.e. P(stem|wholeword). In this case, we would predict the pattern we see: the M170 is largest for the subs items with low whole word frequency and high stem frequency (low P(ww|stem), high P(stem|ww)), where the certainty about the identity of the stem is highest. See Sharpe et al. (2019, p. 138) for a fuller discussion of entropy measures and evoked neural activity. Regardless of direction of correlation, the TP for all prefixed words is correlated with activity in VWFA, suggesting that native Tagalog speakers track morphological units within written prefixed words, irrespective of morphophonological changes. This is the first MEG study to show that decomposition still occurs despite an obscured prefix-stem boundary.

We acknowledge that our study has a relatively small number of stimuli (55 total items) as compared to previous studies that used a single-trial M/EEG analysis. For example, Solomyak and Marantz (2010) had 162 critical items, Fruchter and Marantz (2015) had 200 critical items, and Hauk et al.’s (2006) EEG experiment had 300 total items. However, all these studies investigated general morphological phenomena like suffixation and monomorphemic words. This study is the first to look at less common morphophonological phenomena such as nasal substitution, which resulted in fewer candidate items (see Lewis et al.'s (2011) MEG experiment investigating pseudosuffixation in English with only 78 critical items, and only 11 participants compared to our 18). Future studies should replicate this experiment with more items and participants to see whether the small numerical differences between conditions are significant with larger sample sizes. Regardless, we believe that the combination of MEG and behavioral masked priming data across experiments convincingly show that prefixed words with obscured morphological boundary are decomposed into morphemes.

Successful decomposition in Tagalog stands in contrast to the failure of decomposition in the Hebrew weak root case. One possible reason for this is that Hebrew roots typically consist of just three consonants, so deleting a consonant likely obscures the root identity beyond recoverability and recognizability. In contrast, in Tagalog, root identity is entirely preserved in the case of assimilation, and the *ma-* and *pa-* letter-strings that precede *-m/-n/-ng* may provide a sufficient cue that the word is a prefixed word that may have been nasal-assimilated. The intact root provides the phonological context for such assimilation, supporting this cue. Even in the case of substitution, these initial strings may be a sufficiently robust cue since they reliably occur mostly in the context of prefixation, making decomposition possible and the segmented pieces recoverable. In other words, the loss of information may be less disruptive in Tagalog because the remaining orthographic and phonological information may be used as reliable cues to morphological complexity and decomposability.

Overall, the present study provides both behavioral masked priming and MEG data showing that early, form-based decomposition occurs even when morpheme identity and morpheme boundaries are orthographically and phonologically opaque, and there is variability in the application of morphophonological changes. This is consistent with the literature arguing for decomposition of irregularly inflected forms (Crepaldi et al., 2010; Fruchter et al., 2013), and adds important typological support for models that argue for a decomposition route for all potentially morphologically complex forms (Rastle et al., 2004; Taft & Forster, 1975), regardless of orthographic and phonological changes (McCormick et al., 2008, 2009; Stockall & Marantz 2006; Stockall & Gwilliams, forthcoming). Although none of these visual word recognition models explicitly considered nasal-assimilated and nasal-substituted prefixed words, some may offer different mechanistic accounts of how these words would be decomposed. One example is McCormick et al.’s underspecification-based account (2008, 2009), which argues that orthographic changes can be accommodated by allowing orthographic representations to be underspecified with respect to those aspects of the input that change in certain morphological contexts. For example, the prefix *mang-* and the stem *buhay* “life” would be orthographically represented as {ma_}+{_uhay}, allowing for successful activation of the morphemes when they surface as full forms (e.g., *mang-hula* “to guess,” *habang-buhay* “for life”) and as altered forms (e.g., *mamuhay* “to live”), since there is no mismatch in the orthographic representation and the segmented pieces. In contrast, the readjustment-based account proposes that morphophonological operations are undone in the initial stage of processing while keeping the phonological representation of morphemes fully specified (Stockall & Gwilliams, forthcoming). Based on this proposal, the nasal substitution operation in *mamuhay* ([/maŋ-/➔ [mam-]/_[p, b]) would first be undone during decomposition, activating the vocabulary items {maŋ-}+{buhay}. Our data cannot tease apart which account is more plausible for nasal-assimilated and nasal-substituted prefixed words, but it is also important to note that the accounts of McCormick et al. (2008, 2009) and Stockall and Gwilliams (forthcoming) are not necessarily at odds, since these models consider different levels of representation.

In conclusion, the present findings make theoretical and typological contributions to the literature concerning early, form-based decomposition. Theoretically, morphophonological changes in Tagalog prefixation present a particularly complex processing problem due to the obscured orthographic and phonological boundary they cause and the variability they exhibit. Comparing Tagalog nasal-assimilated and nasal-substituted prefixed words provides an opportunity to finally show that early, form-based decomposition is robust enough to handle prefixed words with obscured morphological boundaries. To the best of our knowledge, Tagalog morphological processing has only been investigated once in psycholinguistics (Cayado et al., 2023) and twice in neurolinguistics (Cayado et al., 2024; Wray et al., 2022), thereby extending the typological breadth of the field. We call for further research looking at other understudied languages with unique morphological phenomena to ensure that our models of morphological processing are not just models of English morphological processing. For example in Bengali, stem access is revealed to be easier within prefixed words than suffixed words due to the fact that stems in prefixation typically remains intact despite morphophonological changes (e.g., assimilation, deletion, resyllabification), while this is not the case in suffixation (see Wynne et al., 2025). Such crosslinguistic investigations help identify possible systematicities and variations in morphological processing, which may eventually result in a more comprehensive model of visual word recognition.

## Supplementary Information

Below is the link to the electronic supplementary material.

Supplementary file1 (DOCX 22 KB)

## Data Availability

The data and experimental materials are available via the Open Science Framework (https://osf.io/zgbrv/).

## Footnotes

1 A neural response peaking approximately 170 ms after the onset of visually presented stimuli originating from the Visual Word Form Area (VWFA; the “Type II activity” in Gwilliams et al., 2016; Tarkiainen et al., 1999).

2 Contrary to *hsryt-HSPYK*, where the initial consonant in the root *srt* is not deleted in the prime /hisrit/ “he filmed.”

3 Though of course the fact that there is such inter-speaker variation at the individual word level for words prefixed with *pang-* and *mang-* plausibly introduces general uncertainty to form-based decomposition for all words with these prefixes.

## Code of availability

All analysis codes are available via the Open Science Framework (https://osf.io/zgbrv/).
