# Supplementary material for: Breaking down prefixed words is unaffected by morphological boundary opacity: Evidence from behavioral and MEG experiments
Source: Psychon Bull Rev. 2025 Aug 12;32(6):3162–75. doi: 10.3758/s13423-025-02758-7 (PMC12627113; doi:10.3758/s13423-025-02758-7)
Supplement: Supplementary file 1 — Supplementary file1 (DOCX 22 KB) [file 13423_2025_2758_MOESM1_ESM.docx]

**Supplementary Materials**

***Experiment 1: Masked Priming Accuracy Data***

Participants showed similar accuracy across prime types in all conditions. This observation is further confirmed by the generalized linear mixed effects model that showed that there was no significant interaction between PrimeType and Condition [*X*^2^(4) *=* 1.40, *p* = 0.6816]. There was also no significant effect of PrimeType [*X*^2^(4) *=* 0.001, *p* = 0.9668], Condition [*X*^2^(4) *=* 2.81, *p* = 0.4204], PrimeLength [*X*^2^(4) *=* 0.55, *p* = 0.455], and TargetFreq [*X*^2^(4) *=* 3.42, *p* = 0.0642].

***Experiment 1: Masked Priming RT Data***

The numerical differences between NoChange and Assim [*estimate* = -2.11, *SE* = 9.51, 95% CI = [-20.75, 16.53], *t* = -0.222, *p* = 0.8246], NoChange and Subs [*estimate* = -6.95, *SE* = 9.47, 95% CI = [-25.53, 11.61], *t* = -0.734 *p* = 0.4627], and Assim and Subs [*estimate* = -4.85, *SE* = 9.53, 95% CI = [-23.55, 13.85], *t* = -0.508, *p* = 0.6111] were not significant.

We further evaluated the numerical difference in priming effects between +Voi and -Voi using a Bayesian parameter approach^[[1]](#footnote-1)^. The Bayesian RT model indicates that the priming effect in + Voi was slightly stronger than in -Voi, but the 95% credible interval (CrI) estimated from the posterior distribution contains a zero (*b =* 14.57, 95% CrI [-10.19, 39.78], R̂ = 1.00), corroborating the frequentist results that the priming effect difference is not credible.

***Experiment 2***

To check for any multicollinearity issues, we tested whether stem:whole word transition probability (TP) and log base frequency (LogBaseFreq) are correlated, since TP is partly derived from base frequency. As shown in Table 1, these two variables were only moderately correlated. We further found that the Variance Inflation Factor (VIF) values for these two variables were lower than the commonly accepted threshold of 5 (TP: VIF = 1.33; LogBaseFreq: VIF = 1.33), indicating no evidence of problematic multicollinearity.

Table 1. Multicorrelations between TP and LogBaseFreq.

| **Variable** | TP | LogBaseFreq |
| --- | --- | --- |
| TP | 1.000 | -0.501 |
| LogBaseFreq | -0.501 | 1.000 |

For reaction time, the Subs condition has the highest RT (1145ms), followed by NoChange (1131ms), then Assim (1110ms). Mixed-effects modelling (see Table 2 for the full model) revealed no significant interaction between Condition and TP [*X*^2^(4) = 1.973, *p* = 0.372], but there was a significant effect of TP [*X*^2^(4) *=* 10.119, *p* = 0.001] and LogBaseFreq [*X*^2^(4) *=* 7.741, *p* = 0.005]. There was no significant effect of Condition, [*X*^2^(4) *=* 2.158, *p* = 0.339].

Table 2. Reaction time mixed-effects model summary

| **Formula:** RT ~ Condition * TP + LogBaseFreq + (1\|Participant) + (1\|Item) | | | | |
| --- | --- | --- | --- | --- |
| **Fixed effects:** | **Estimate** | ***df*** | ***t* value** | **Pr(>\|*t*\|)** |
| (intercept) | 1275.889 | 62.719 | 18.947 | <.0001*** |
| Condition = Assim | 8.229 | 51.247 | 0.196 | 0.845 |
| Condition = Subs | 32.079 | 50.841 | 0.725 | 0.471 |
| TP | -1078.923 | 50.630 | -0.982 | 0.330 |
| LogbaseFreq | -67.307 | 49.447 | -2.741 | 0.008* |
| Interaction, TP:Condition = Assim | -1948.523 | 50.786 | -1.411 | 0.164 |
| Interaction, TP:Condition = Subs | -978.764 | 49.938 | -0.671 | 0.505 |
| Significant codes: 0 ‘***’ 0.001 ‘**’ 0.01 ‘*’ 0.05 ‘.’ 0.1 ‘ ’ 1 | | | | |
| **Random effects:** | **Variance** |  |  |  |
| Participant | 29363 |  |  |  |
| Item | 5060 |  |  |  |
| Residual | 53486 |  |  |  |

For accuracy, the generalized mixed-effects model with the same fixed effects and random-effect structure as RT data revealed no significant interaction between Condition and TP [*X*^2^(4) = 1.447, *p* = 0.484], but there was a significant effect of TP [*X*^2^(4) = 3.880, *p* = 0.048]. There were no significant effects of LogBaseFreq [*X*^2^(4) = 2.395, *p* = 0.121] and Condition [*X*^2^(4) = 0.715, *p* = 0.699].

1. We fitted Bayesian hierarchical linear models to RT as a function of StemC (reference level “-Voi”), and PrimeType (reference level “Related”) and interaction, using the Stan modelling language (Carpenter et al., 2016) and the package *brms* (Buerkner, 2016). We included by-participant and by-item random intercepts. We used the default priors suggested by *brms* except for the coefficients for the PrimeTypeUnrelated and the interaction, for which we applied weakly informative priors of β ∼ *N*(0, 100) in order to rule out improbably large effect sizes. Four sampling chains ran for 2000 iterations with a warm-up period of 1000 iterations for each model, thereby yielding 4000 samples for each parameter tuple. =An effect is credible if the 95% credible interval (CI) estimated from the posterior distribution does not contain zero (see Angele et al., 2022). [↑](#footnote-ref-1)
